# Recurrent Urinary Tract Infection: A Mystery in Search of Better Model Systems

**DOI:** 10.3389/fcimb.2021.691210

**Published:** 2021-05-26

**Authors:** Benjamin O. Murray, Carlos Flores, Corin Williams, Deborah A. Flusberg, Elizabeth E. Marr, Karolina M. Kwiatkowska, Joseph L. Charest, Brett C. Isenberg, Jennifer L. Rohn

**Affiliations:** ^1^ Centre for Urological Biology, Department of Renal Medicine, University College London, London, United Kingdom; ^2^ Department of Bioengineering, Charles Stark Draper Laboratory, Inc., Cambridge, MA, United States

**Keywords:** urinary tract infection (UTI), microphysiological systems, *in vitro* infection model systems, organ-on-chip, organoid, urothelium, uropathogenic *E. coli* (UPEC), mouse models

## Abstract

Urinary tract infections (UTIs) are among the most common infectious diseases worldwide but are significantly understudied. Uropathogenic *E. coli* (UPEC) accounts for a significant proportion of UTI, but a large number of other species can infect the urinary tract, each of which will have unique host-pathogen interactions with the bladder environment. Given the substantial economic burden of UTI and its increasing antibiotic resistance, there is an urgent need to better understand UTI pathophysiology – especially its tendency to relapse and recur. Most models developed to date use murine infection; few human-relevant models exist. Of these, the majority of *in vitro* UTI models have utilized cells in static culture, but UTI needs to be studied in the context of the unique aspects of the bladder’s biophysical environment (e.g., tissue architecture, urine, fluid flow, and stretch). In this review, we summarize the complexities of recurrent UTI, critically assess current infection models and discuss potential improvements. More advanced human cell-based *in vitro* models have the potential to enable a better understanding of the etiology of UTI disease and to provide a complementary platform alongside animals for drug screening and the search for better treatments.

## Introduction: Urinary Tract Infection – A Globally Important Disease in Need of Better Human-Based Model Systems

UTIs are among the most common infectious diseases worldwide, causing approximately 150 million cases per annum (Stamm and Norrby, 2001), but are significantly understudied (Losada et al., 2016). Although much more common in women, UTI can affect men and children (Foxman, 2010) and after respiratory infection, it is the most common infectious disease of our ageing population (Rowe and Juthani-Mehta, 2013). In care homes, UTI is the most common infectious disease of all (Rowe and Juthani-Mehta, 2013). What’s more, UTI is one of the most frequent healthcare-acquired infections, and it is particularly problematic for people with multiple sclerosis, spinal injuries, pregnant women and patients requiring urinary catheters (Foxman, 2003). As a result, UTI imposes a substantial economic and healthcare burden (Foxman, 2010). In addition, because of the vast number of individual treatments required, the World Health Organization has described antibiotic resistance in uropathogens as a key pressure point in the growing global antimicrobial resistance crisis (WHO, 2014). As a number of the bacteria involved also infect different bodily niches, resistance can affect treatment of other diseases, including those requiring surgery. There is therefore an urgent need to better understand UTI pathophysiology, so that alternatives to antibiotics can be developed.

Uropathogenic *E. coli* (UPEC) accounts for about 80% of community-acquired UTI in otherwise healthy people (Foxman, 2003), but a large number of other bacterial species can cause a UTI (Foxman, 2010). One of the biggest concerns about UTI is its tendency to recur. While there have been advances in new treatments and vaccines (reviewed in O’Brien et al., 2016), antibiotics remain the mainstay of therapy. Even despite treatment, up to 25% of women experience a relapse within six months (Foxman et al., 2000); in one large study, 2% had six or more episodes within a two-year period (Laupland et al., 2007). Some patients experience recurrent UTI for years, necessitating prophylactic antibiotics that only increase the risk of antimicrobial resistance (Selekman et al., 2018). Although the pathophysiology of UTI – primarily of UPEC-associated disease – has been extensively researched in animal models (Hunstad and Justice, 2010), relatively little is understood about the infection cycle in humans. In particular, the behavior of *E. coli* pathogens in the human bladder lumen is understudied, and the situation for other bacterial species is even hazier.

To develop new treatment strategies for UTI, it is imperative that researchers understand the pathophysiology. Although careful studies in UTI patients have been greatly illustrative, as with most diseases, there are limits to what can be understood in this context. The study of UTI with small animal models and cell culture systems, while also incredibly valuable, still have limitations. Further progress in improving the lot of UTI patients will require advances in human-based model systems.

In this review, we will discuss the proposed mechanisms of UTI pathogenesis and recurrence, and highlight the unknowns that remain. Next, we will discuss the animal models used to study UTI, followed by the main *in vitro* human-cell-based models, examining their strengths and limitations. Then we will review the key features of human bladder physiology that ideally would be present in an improved *in vitro* model system. Finally, we will discuss state-of-the-art platforms and solutions to these challenges, and conclude with our perspectives on where the field is headed.

## The Mechanisms of Recurrent UTI: A Multi-Faceted Problem

Recurrent urinary tract infection (rUTI) represents a massive burden for the economy, for healthcare systems and for the patients who suffer from them. Several hypotheses for the mechanism of rUTI have emerged over the past decades – which are not necessarily mutually exclusive. Indeed, different – and likely multiple – scenarios will exist within different patients and across different species of uropathogen, as shown recently (Thänert et al., 2019). We argue that better human-based infection models will be required to elucidate them fully.

### Microbiota, Dysbiosis, and Distal Reservoirs

One of the most long-standing theories for recurrence of UTI is that the gastrointestinal tract functions as a reservoir for uropathogens, which are repeatedly reintroduced into the urinary tract *via* contamination of the periurethral surface and subsequent retrograde ascension. Indeed, common uropathogens are abundant as gut commensals, and there are many papers supporting this mechanism (reviewed recently in (Jones-Freeman et al., 2021). In agreement, recent studies with patients experiencing rUTI revealed that re-infection was usually preceded by a bloom of uropathogens in the intestine (Thänert et al., 2019), and that gut abundance of *Escherichia* and *Enterococcus* served as an independent risk factor for late problematic urine colonization in kidney transplant recipients, with genomic strain analysis supporting the association (Magruder et al., 2019). Also in support of this hypothesis, molecules designed to selectively deplete uropathogenic *E. coli* in the gut also decrease the incidence of UTIs in mice [e.g. (Spaulding et al., 2017)].

The gut is not the only proximal niche that might harbor uropathogens, however. A crosstalk between the vaginal and urinary microbiomes has been reported based on both clinical correlations and experimental models, and the data strongly suggest that this interconnection affects the recurrence of UTIs (Komesu et al., 2020; Lewis and Gilbert, 2020). Moreover, the now well-established observation that healthy urinary tracts are not sterile and that their microbiota is distinct from those of chronic UTI patients has highlighted the potential relationship between bladder dysbiosis and rUTI (Khasriya et al., 2013; Hilt et al., 2014; Neugent et al., 2020). Dysbiosis, defined as an imbalance in the natural microbial community, may even affect several host protective mechanisms, such as those mediated by commensals. Also, alteration of the urinary microbiota through the introduction of native microbes from either the gut or the vagina may potentiate the recurrence of UTI. For example, the vaginal commensal *Gardnerella vaginalis* was reported in the urinary tract of both human males and females (Fairley and Birch, 1983; Lam et al., 1988). More recently, the inoculation of *Gardnerella* into the bladders of mice that had recovered from UPEC infection caused urothelium exfoliation and facilitated the exposure of intracellular UPEC reservoirs in the bladder (Gilbert et al., 2017). In addition, this exposure increased the severity of infection compared with control mice. Of note, a small clinical trial showed that the introduction of a probiotic Lactobacillus species capable of outcompeting uropathogens into the vagina of women with rUTI was able to modestly reduce the incidence of UTI (Stapleton et al., 2011). Reciprocally, commensal *Lactobacillus* in the vagina establish a low pH that is non-permissive to uropathogenic species such as *E. coli*; if these commensals are outcompeted, uropathogens can become the dominant vaginal species, which would facilitate their eventual transit to the urethra (Brannon et al., 2020; Lewis and Gilbert, 2020).

Antibiotics are another important factor affecting rUTI, not only those taken to treat UTI, but also other indications. Antibiotics favor the development and proliferation of multidrug-resistant organisms (either in the bladder or in the gut/vagina), as well as increase the availability of niches that are no longer inhabited by commensals. As a result, these niches can become dysbiotic and more easily colonized by more virulent and persistent bacteria (Chen et al., 2013).

Taken together, these various strands of evidence have boosted the search for probiotic approaches and other therapies focused on the modulation of microbiota in the UTI context. However, a human cell-based model that could accurately mimic the human urinary microbiota and its interactions with other microbiota would be a very helpful tool in this endeavor.

### Bacterial Virulence and Bladder Reservoirs

Many strain-specific bacterial virulence factors may contribute to the recurrence of UTI, such as flagella/pili, adhesins, extracellular polysaccharides, lipopolysaccharides, toxins, ureases, proteases and iron-scavenging siderophores. These factors allow uropathogens to survive during long periods in a nutrient-limited habitat, helping them to adhere, colonize, damage and invade host cells, as well as to evade host defenses, ultimately increasing their persistence in the urinary tract. For instance, a common uropathogenic strategy is the formation of biofilms, either directly on the urothelial surface or on indwelling devices such as catheters (Jansen et al., 2004; Jacobsen and Shirtliff, 2011). This behavior has been reported in *Klebsiella pneumoniae* (Reid et al., 1992; Stahlhut et al., 2012), *Pseudomonas aeruginosa* (Ivanova et al., 2015; Saini et al., 2015), UPEC (Ferrières et al., 2007) and Enterococci (Sillanpää et al., 2010), and may even be facilitated by polymicrobial interactions during infection (Gaston et al., 2020). The biofilm provides an ideal physicochemical barrier that protects uropathogens from antimicrobial agents, host immunity and other stresses, allowing them to persist and reinfect the urinary tract. Furthermore, a subpopulation of bacterial cells in biofilms, the so-called *persistors*, are known to reversibly reduce their metabolic activity, adopting a dormant state that can evade host defenses as well as treatments that target active metabolic pathways or activities such as cell division (Wood et al., 2013). Another strategy is the adhesion of bacteria either to the host cell surface and/or the extracellular matrix (ECM), through the use of a vast arsenal of virulence factors. Among the most common are curli proteins (similar to amyloid fibers), which are major facilitators of UPEC colonization (Luna-Pineda et al., 2019). These proteins allow bacterial binding to ECM and serum proteins such as fibronectin, laminin and plasminogen, and are highly expressed among Enterobacteriaceae. P-type pilus and the FimH fimbrial adhesin (part of the Type-1 pilus) have also received attention due to their high binding affinity with urothelial receptors, being important players for UPEC colonization and/or invasion in several UTI models (Mulvey et al., 1998; Zhou et al., 2001; Bishop et al., 2007; Rosen et al., 2008; Feenstra et al., 2017). Therefore, a number of therapeutic approaches against rUTI target specific virulence factors, mainly blocking adhesion and/or biofilm formation (Liao et al., 2012; Flores-Mireles et al., 2014; Mike et al., 2016).

Another bacterial mechanism implicated in rUTI is the ability of bacteria to invade host urothelial cells and establish intracellular bacterial communities (IBCs). This was first discovered in the early 2000s in a UPEC mouse model of induced UTI, in which the bacteria subverted host defenses, invaded urothelial cells and formed IBCs that could later erupt and re-establish UTI (Anderson et al., 2003; Justice et al., 2004). In this exciting model, after invasion, bacteria rapidly multiply in the cytoplasm of superficial bladder epithelial cells, where they form “pods” that can expand, blister-like, into the lumen of the bladder as the community grows (Anderson et al., 2003). These communities are embedded in a biofilm-like matrix, which may confer a protective effect similar to that seen in more conventional biofilms. In other cases, UPEC may even invade deeper layers of the urothelium, remaining in a membrane-bound compartment with little to no metabolic activity. These “quiescent intracellular reservoirs” (QIR) can be latent for months in mice, and can be re-activated after the exfoliation of the upper urothelium cell layers (Mulvey et al., 2001; Mysorekar and Hultgren, 2006).

While IBCs have also been experimentally demonstrated in human cancer cell lines (e.g. (Bishop et al., 2007; González et al., 2020), nearly two decades on from the original IBC discovery in mice, surprisingly few papers have reported the existence of UPEC IBCs in patients. A handful show IBC using exfoliated urothelial cells from the urine of human UTI patients (Rosen et al., 2007; Robino et al., 2013; Robino et al., 2014; Cheng et al., 2016), although only some (Robino et al., 2013; Robino et al., 2014) used imaging resolution with sufficient discriminatory power to distinguish intracellular bacteria from those on the surface of these notoriously flat cells. To our knowledge, only one group has studied human biopsies and reported the existence of IBC and QIR (De Nisco et al., 2019). In contrast, in a recent study with a porcine model, which has a more similar urogenital anatomy, physiology and immune response to human compared with small-animal models, IBCs were not observed in the bladder after UPEC infection, although high loads of bacteria were detected after prolonged infection, some forming biofilm-like extracellular aggregates (Nielsen et al., 2019). Therefore, more studies on the role of IBC and QIR in rUTI in humans or human bladder model systems would be welcome.

Although intracellular lifestyle stages have been observed mainly in UPEC infection, other uropathogens, such as *Klebsiella pneumoniae* (Rosen et al., 2008), *Staphylococcus saprophyticus* (Szabados et al., 2008) and *Salmonella enterica* (Bishop et al., 2007) might also display them, at least in murine models and/or cell lines. More recently, *Enterococcus faecalis* was shown to reside inside urothelial cells shed from patients with chronic UTI (Horsley et al., 2013), and to invade a human organoid model (Horsley et al., 2018). It remains to be seen whether other uropathogens invade human cells, and how widespread the phenomenon is in patients.

### Morphological Alterations

Recurrent infection may also be facilitated by the ability of some uropathogens to alter their morphology to evade the host immune system and recolonize naïve regions in the urinary tract more easily. One well-studied example is UPEC cell filamentation, a process which occurs when bacterial cells emerge from IBCs (Justice et al., 2006). This filamentous form confers resistance to phagocytic engulfment (Justice et al., 2006) and may provide enhanced adhesive properties (Andersen et al., 2012). Another example is *Proteus mirabilis*, which can increase flagellar density (Armbruster and Mobley, 2012). Recently, Mickiewicz et al. reported that UPEC may also acquire a reversible “L-form”, a cell-wall deficient phase that can evade antibiotic treatments targeting the bacterial cell wall. In both urine and a zebrafish model, UPEC could rapidly switch into this form; the L-form was also the most prevalent in urine from older rUTI patients (Mickiewicz et al., 2019).

### Bacterial Resistance and Resilience

Antibiotic resistance is a key issue in recurrent UTI, and is the most well-studied mode of treatment failure generally (Ho et al., 2019; Yelin et al., 2019; Mattoo and Asmar, 2020). The overuse of antibiotics has positively selected for strains with specific genetic traits allowing them to survive and proliferate in the presence of a single or even a class of antimicrobial compounds. In addition, most of these bacteria have the necessary machinery for intra- and inter-species transmission (e.g. through horizontal gene transfer mechanisms), which can spread the selected traits rapidly and boost the generation of multidrug-resistant uropathogens (Ho et al., 2019; Mattoo and Asmar, 2020).

On the other hand, a much less well-understood phenomenon involves resilience behaviors that provide temporary antibiotic evasion in a sub-population of bacterial cells. The emergence of resilient bacteria in an overall susceptible bacterial population might therefore play an important role in the selection of fitter uropathogens and subsequent recurrence. Several genetic and non-genetic mechanisms might be involved in the development of resilient profiles, which can be generally categorized as tolerance, persistence and heteroresistance phenotypes (Carvalho et al., 2019).

One of the best studied bacterial resilient mechanisms involved in rUTI are biofilms, which provide tolerance to external stresses, such as antibiotic treatments and host defenses (Olsen, 2015). Bacteria in polymicrobial UTI also have the ability to protect one other from clinically relevant antibiotics through the increase of tolerant/resilient phenotypes in the bacterial community (de Vos et al., 2017). In contrast, the mechanisms of persistence and heteroresistance are still largely unexplored in the UTI context, as well as their impact in clinical settings. However, the development of bacterial subpopulations that are dormant/less metabolically active and/or display a heterogenous (more resistant) phenotype might be crucial for the success of a pathogenic community facing inconsistent and unexpected environmental challenges (Kussell et al., 2005; Carvalho et al., 2019). Although much remains to be learned about this interesting class of treatment failure, bacterial resilience may have a role in the development of chronic UTIs, while resistance may play a more prominent role in recurrent acute infections (Olsen, 2015). It is important to note that the host context is likely to influence bacterial resilience phenotypes, so studying this phenomenon in a human-cell (and one day, patient-specific) environment will be important.

### Host Factors

Bacteria are not the only players to alter their properties during the host/pathogen interaction. Host urothelial cells can also change upon first infection, a phenomenon observed in experimental mouse models of chronic UPEC infection and in cell lines. These changes influence host cell gene expression, shape, size, growth and proliferation, and depending upon what is changed, may make the urothelium more resilient, or more susceptible, to re-infection (Mysorekar et al., 2009; Shin et al., 2011; O’Brien et al., 2016).

Other host factors, mainly immune-related, also play a prominent role in the chronicity of rUTI, as the recurrence of infection is frequently associated with a disturbed innate immune response and/or insufficient adaptive immunity (recently reviewed in (Lacerda Mariano and Ingersoll, 2020). The reasons behind these events are still unclear and largely unexplored due to the lack of knowledge about how the immune response is triggered and develops in human UTIs, which can differ significantly from the murine context. Indeed, up until recently it was assumed that adaptive immunity was not involved at all, due to early cumulative evidence from immune-deficient mouse models lacking interleukins, immunoglobulins and/or T lymphocytes, that could nevertheless resolve UTI as well as their wild-type counterparts or even be resistant to infection (Svanborg Edén et al., 1984; Ragnarsdóttir et al., 2008). A revision of this view has paved the way for the development of vaccines against UTI, some of which are showing promising results (Prattley et al., 2020).

It has been increasingly reported, mainly in children, that a genetic component might be involved, as polymorphisms and mutations in innate immunity-related genes can increase susceptibility to UTIs (Karoly et al., 2007; Tabel et al., 2007). Additionally, an impaired immune response (Thumbikat et al., 2006) as well as an exacerbated pro-inflammatory response to a primary infection (Hannan et al., 2014), might favor recurrence of infection. In fact, the activation of host immunity frequently leads to severe injuries in the urinary mucosa/urothelium, which become more prone to subsequent infections. In one way or another, this common suboptimal and non-sterilizing immune response may create the perfect environment for the occurrence of rUTIs in an experienced host.

## Animal Models for Studying UTI

### Brief History of Animals in UTI Research

Animals of many species were essential for biological scientific experiments as early as the late 19^th^ century, furthering our understanding of physiology and human diseases. The earliest mention of establishing infection in animal bladders was in 1873, when Fels and Ritter inoculated canine bladders to induce cystitis using urethral ligation (Keyes, 1894). Many tried to induce infection in animals using pure cultures of microorganisms, but ligation or a wound to the bladder was always necessary to establish cystitis. In 1890, Schnitzler managed to induce cystitis in rabbits without ligation using ‘Urobacillus liquefaciens septicus’ [later defined as a member of the *Bacillus cloacae* group (Archer, 1931)]. Later, it was reported that ‘coliform bacillus’ could produce cystitis in rabbit kidneys and an inflammatory response in the bladder mucosa (Lepper, 1921). Animals continued to be important for UTI research, mainly rats and rabbits, until the first murine model of UTI was described in 1967, as an experimental infection model for pyelonephritis (Keane and Freedman, 1967).

Murine models eventually became established as a valuable tool to study UTI, a trend which continues to this day. Mice were considered superior to rats because they were slightly more relevant to humans, with a greater abundance of globoseries glycolipid receptors on urothelial cells for attachment (Hagberg et al., 1983). Similar to humans, mice do not have a natural vesicoureteral reflux, unlike other rodent models, so would be a more physiologically accurate model for pyelonephritis (Hvidberg et al., 2000). Mouse and human bladders also have highly conserved uroplakins, which aid type 1 fimbriae adherence and invasion by UPEC (Zhou et al., 2001). Recently, female C57BL/6 mice urothelial cells were analyzed and classified into eight clusters dependent on expression of cell-specific markers. A novel urothelial cell type expressing Plxna4 was discovered in the mouse bladder (Li et al., 2021) which is conserved in humans and may play a role in host immune response (Wen et al., 2010).

As mice do not naturally develop urinary infections, instillation techniques were developed. Hagberg et al. described the ascending, unobstructed UTI instillation in female CBA mice with *E. coli* (Hagberg et al., 1983) which has since been adapted for other uropathogens such as *Proteus mirabilis* and *Enterococcus faecalis* (Jones et al., 1990; Shankar et al., 2001). This instillation method helped replicate the attachment of human-derived UTI isolates to murine urothelial cells which was considered essential for understanding human infection. CBA mice were preferred due to better bacterial attachment and colonization; female CBA were favored as the anatomy of male mice presented challenges for urethral inoculation (Hagberg et al., 1983). Although UTI is more prevalent in females than males (Foxman, 2010), little is known about sex differences in UTI pathophysiology. To address this, a protocol for inducing UTI in male mice using transurethral catheterization allowed direct comparison between male and female C57B1/6 mice and their host response (Zychlinsky Scharff et al., 2017). However, all instillations *via* catheterization bypass the biology of ascending UTI *via* the urethra (Barber et al., 2016).

Many different mouse models have been used for UTI (**Table 1**). The mouse model offers systemic context and a diverse range of genetic variability, allowing researchers to test specific host factors and immune responses in transgenic and knockdown mutants (Barber et al., 2016). ‘Germ free’ murine models are also potentially an attractive option for modeling UTI and the gut microbiota/UTI axis, allowing exclusive colonization of certain pathogens or purposefully introduced commensal species. For immunological purposes, naturalizing mouse models can also be used to help translate research to humans (Graham, 2021). The immune profiles of laboratory mice are different from that of human as they have a low density of mature T cells (Beura et al., 2016) and lower LPS sensitivity which could lead to differences in pathogenesis and treatment responses in humans (Graham, 2021). However, germ-free mice are more expensive and need specialized equipment and training (Kennedy et al., 2018). To our knowledge, however, there have been no reports of naturalized mice used for UTI research.

**Table 1:** Mouse models used for UTI research.

Mouse strain	Strain characteristics	UTI work and findings
C57BL/6	Used in most studies (Timmermanset al., 2017)	8- and 12-weeks post infection with *Enterococcus faecalis*, C57BL/6 mice had a lower incidence of infected kidneys than SWR mice but a higher proportion of infected kidneys by week 8 compared with mouse strain A (Guze et al., 1985)
	Due to their popularity not all C57BL/6 strains are the same with differences between C57BL/6J and C57BL/6N strains (Simon et al., 2013; Kelmenson, 2016)	Demonstrated extremely low inflammation of the kidneys throughout the 14 day infection with *E. coli* 1677 (Hopkins et al., 1998)
		Infected with either *E. coli* WT NU14 or its fimH^−^ analogue. 6h post-infection, some WT *E. coli* formed microcolonies in the bladder, while some adhered individually to the surface; fimH^−^ bacteria did not attach (Mulvey et al., 1998)
		75% of infected bladders remained colonized with a *fimH* isogenic mutant of *E. coli* 2 weeks post-infection (Mulvey et al., 2001)
		Infected with *E. coli* UTI89 and CFT073. Intracellular bacterial communities (IBCs) present 6h post infection. IBCs in C57BL/6J occasionally had more loosely defined edges (Garofalo et al., 2007)
		Infected with *E. coli* asymptomatic bacteriuria strain VR50; after 18h, VR50 could colonize the urothelium but not mutants for afimbrial adhesin (Beatson et al., 2015)
		Infected with *E. coli* asymptomatic bacteriuria strain 83972 which ameliorated the effects of a 24h challenge by a UPEC strain (Rudick et al., 2014)
CBA	Large kidneys with proneness to tubulointerstitial lesions (Rudofsky, 1978)	Evidence of virulence factor type 1 fimbriae in *E. coli* (Gunther et al., 2001)
	Greater abundance of globoseries glycolipid receptors on urothelial cells for attachment of UTI pathogens compared to humans (Hagberg et al., 1983)	Infected with *E. coli* UTI89 and CFT073. IBCs present 6h post infection. Similar IBC morphology compared with other mouse strains (Garofalo et al., 2007)
	More susceptible to UTI infection than BALB/c (Hagberg et al., 1983), C57 and C3H/HeN mice (Hagberg et al., 1983)	40 CBA/J mice were infected with *E. coli* CFT073. 313 bacterial genes were upregulated and 207 downregulated. Chemotaxis- and flagella motility-associated genes were downregulated *in vivo* compared to *in vitro* (Snyder et al., 2004)
CF1	Albino white mice	11 different *E. coli* strains were analyzed for infectivity. Presence of C175-94 strain in bladder was found in 4/6 mice 4 weeks post-infection (Hvidberg et al., 2000)
	Outbred to Charles River Laboratories in 1974 (Chia et al., 2005)	
	Have been used for EGFR gene knockout studies so can be genetically manipulated (Threadgill et al., 1995)	
FVB/NJ	Nephrotic syndrome-like characteristics: albuminuria, cholesterolemia, predisposition to increased T2 cell response; no autoimmune origin (Maier et al., 2007)	Infected with *E. coli* UTI89 and CFT073. IBCs present 6 hours post infection. Similar IBC morphology with other mouse strains (Garofalo et al., 2007)
	Ccr5^P185L^ mutation results in resistance to paracetamol (Timmermans et al., 2017)	
	Mx1^s1^ results in susceptibility to myxoviruses such as Influenza (Grimm et al., 2007)	
	High susceptibility to glomerular disease (Uchio-Yamada et al., 2016)	
	Used in autoimmunity research due to proneness to develop asthma-like disease with high levels of IgE (Zhu and Gilmour, 2009)	
C3H/HeJ	IL1a^Y118_T119del^ results in resistance to IL1α-mediated inflammation (Timmermans et al., 2017)	Clears LPS-containing Gram-negative bacteria at a slower rate compared with C3H/HeN mice. There was no difference for clearing Gram-positive bacteria such as *Staphylococcus saprophyticus* (Hagberg et al., 1984)
	Tlr4^P712H^ (missense mutation) results in resistance to LPS-induced shock (Timmermans et al., 2017)	When infected with *E. coli* 1677 (O6, type 1), mice had increased bacterial growth in the bladder after 3 days. Inflammation response to LPS was poor and only slightly increased from Day 3 to day 14 post infection (Hopkins et al., 1996)
	LPS Resistant (HeJ^-^) (Tötemeyer et al., 2003)	Infected with *E. coli* CI5 to develop prolonged UTI in the bladder. Assessed Forskolin as a potential treatment for UTI (increases cAMP-driven exocytosis). Multiple intraperitoneal injections of 10mg/kg Forskolin at 6, 24 and 48 hours post infection reduced colonization of intracellular *E. coli* (Bishop et al., 2007)
	C3H mice have mutation in *Nramp1* G169D that makes them susceptible to intracellular pathogens like Mycobacterium, Salmonella & Leishmania (Vidal et al., 1996)	Infected with *E. coli* UTI89 and CFT073. IBCs present 6h post infection. Similar IBC morphology compared with other mouse strains (Garofalo et al., 2007)
		Filamentation of *E. coli* UTI89 in C3H/HeJ mice is observed only in the later stages of infection. Could be due to lack of functional TLR4 and resulting lack of inflammatory response (Justice et al., 2006)
C3H/OuJ	C3H mice have mutation in *Nramp1* G169D that makes them susceptible to intracellular pathogens like Mycobacterium, Salmonella & Leishmania (Vidal et al., 1996)	When infected with *E. coli* 1677 (O6, type 1), infection became more severe with increased bacterial growth. Unlike other strains in which inflammation gradually decreased, C3H/OuJ had increased inflammation in both, kidney and bladder (Hopkins et al., 1998)
	LPS sensitive (Hopkins et al., 1996)	
C3H/HeN	IL1a^Y118_T119del^ results in resistance to IL1α-mediated inflammation (Timmermans et al., 2017)	Cleared infection of LPS-containing Gram-negative bacteria faster than C3H/HeJ mice but not Gram-positive bacteria *Staphylococcus saprophyticus* (Hagberg et al., 1984)
	C3H mice have mutation in *Nramp1* G169D that makes them susceptible to intracellular pathogens like Mycobacterium, Salmonella & Leishmania (Vidal et al., 1996)	Infected with *E. coli* 1677 (O6, type 1). Demonstrated a high level of infection in the bladder over 14 days compared with other mouse strains (Hopkins et al., 1998)
		Had a significantly reduced bacterial load when infected with *E. coli* 1677 than C3H/HeJ and C3H/OuJ over 14 days (Hopkins et al., 1996)
		Infected with *E. coli* UTI89 and CFT073. IBCs present 6h post infection. Similar IBC morphology compared with other mouse strains (Garofalo et al., 2007)
		Infected with *E. coli* UTI89 or *K. pneumoniae* TOP52 FimH strain, WT or genetically engineered fimH knockouts. WT-infected bladders had significantly higher CFU counts in *E. coli* at 6h, 24h and 336h post-infection. In contrast, WT *K. pneumoniae* showed no significant difference in bacterial count at 6h and 24h post-infection and became significantly lower at 336h post-infection. *K. pneumoniae* fimH was important for intracellular bacterial colonization (Rosen et al., 2008)
		Infected with *E. coli* UTI89 using the technique described in (Olson et al., 2016). Mice were treated with ceftriaxone, but a few mice were left with residual UPEC. When infection cleared, kidneys of treated mice revealed to have multiple scars containing cellular infiltrate. Males, but not females, can develop 100% penetrant robust ascending UTI presenting with renal abscesses and fibrosis, and fail to resolve it (Olson et al., 2017)
DBA/1 & DBA/2	DBA/1 are susceptible to immune mediated nephritis (Xie et al., 2004)	Infected with *E. coli* 1677 (O6, type 1), DBA/1 and DBA/2 strains could resolve the initially high level of infection in bladder and kidney (Hopkins et al., 1998)
	DBA/2 lack surface expression of CD94/NKG2A on NK cells known to be expressed on most fetal NK cells (Vance et al., 2002)	DBA/1 mice were infected with *Pseudomonas aeruginosa* for phage therapy applications and found there was more enhanced killing of intracellular bacteria (reviewed in (Cieślik et al., 2021)
BALB/c	Naturally resistant to prolonged UTI (Bishop et al., 2007)	8- and 12-weeks post infection with *S. faecalis* GK (ATCC 23241), BALB/c mice had a significantly lower incidence of infected kidneys compared with SWR mice (Guze et al., 1985)
	Used in cancer research and when older can develop renal tumors in males (Noronha, 1977)	Infected with *E. coli* 1677 (O6, type 1), BALB/c mice had highest bacterial presence in the bladder and second highest in the kidneys after 24 hours. After 14 days, infection decreased in both bladder and kidney (Hopkins et al., 1998)
		Infected with *E. coli* CI5 to determine the efficacy of Forskolin as a treatment for UTI (increases cAMP-driven exocytosis). Intravesical 100µM Forskolin reduced colonization of intracellular *E. coli* by more than 75% compared to saline controls (Bishop et al., 2007)
AKR	Mainly used in cancer research and immunology research to produce theta AKR antigen (EMBL-EBI, 2021)	After infection with *S. faecalis* GK (ATCC 23241), AKR mice had established pyelonephritis after 8 weeks and had the second highest bacterial load after 12 weeks in kidneys (Guze et al., 1985)
		Infected with *E. coli* 1677 (O6, type 1), AKR mice could resolve the initially high level of infection in the kidneys after 5-7 days but not in the bladder (Hopkins et al., 1998)
SJL	Susceptible to experimental autoimmune encephalomyelitis useful for multiple sclerosis research (reviewed in (Croxford et al., 2011)	SJL mice had a significantly higher proportion of infected kidneys than Strain A at week 8 and similar to other mice (Guze et al., 1985)
	Elevated T-cell level (Jain et al., 2015)	Infected with *E. coli* 1677 (O6, type 1), SJL strains showed a very low but constant level of infection in bladder and kidney (Hopkins et al., 1998)
SWR	Develop very severe polydipsia and pyuria as a consequence of nephrogenic diabetes insipidus (Kutscher and Miller, 1974)	When infected with *S. faecalis* GK (ATCC 23241), SWR mice had increased ascending UTI in the kidneys compared to Strain A mice. No histological changes to the kidney were seen and the infection was cleared effectively. When infected with *E. coli* strain Yale, SWR mice had a significantly increased bacterial load in the kidneys with more histopathological changes than Strain A mice over 12 weeks (Guze et al., 1987)
	Kidneys unresponsive to vasopressin (Kutscher et al., 1975)	24h post infection with *S. faecalis* GK (ATCC 23241), SWR mice had increased bacterial growth compared to strain A mice. Cortical abscesses and scars at week 1 and 4 respectively were significantly greater in SWR mice than strain A mice. (Guze et al., 1985)
	SWR/J are resistant to obesity on high fat diets (Leibowitz et al., 2005)	Infected with *E. coli* 1677 (O6, type 1), SWR mice showed very low but constant level of infection (Hopkins et al., 1998)
A	Suffer from specific complement deficiency (Timmermans et al., 2017)	When infected with *S. faecalis* GK (ATCC 23241), Strain A mice exhibited lower kidney infection with no significant histopathological changes compared with SWR mice. Infection was cleared effectively for both.
	Macrophage defects (Timmermans et al., 2017)	Within 12 weeks post infection with *E. coli* strain Yale, Strain A mice had a significantly decreased bacterial count in the kidney and no histopathological changes. (Guze et al., 1987)
		When infected with *S. faecalis* GK (ATCC 23241) cortical abscesses and scars were significantly reduced after 1 week in strain A mice compared to SWR mice. Strain A had a significantly lower proportion of infected kidneys than all other tested mouse strains. (Guze et al., 1985)

### Differences Between Mouse and Human Models

Although murine models remain incredibly valuable for UTI, they are expensive and labor-intensive to maintain and breed. This precludes their use for high-throughput analysis, particularly for drug screening and testing. Ethical guidelines and applications have also slowed down animal research, although scientists can navigate these obstacles with a little patience.

Aside from these logistical issues, there is a deeper concern that animal models do not always recapitulate the human environment well enough to predict how disease physiology works, nor how prospective treatments might behave in human patients (Herati and Wherry, 2018), especially as mice do not naturally acquire UTI. Accordingly, the use and accuracy of animal models is a frequent discussion point among UTI researchers, with Barber et al. reviewing their strengths and limitations (Barber et al., 2016). Since then, further questions about the accuracy of animal models, especially mice, have arisen.

#### Host Bladder Physiological Differences

**Figure 1:**
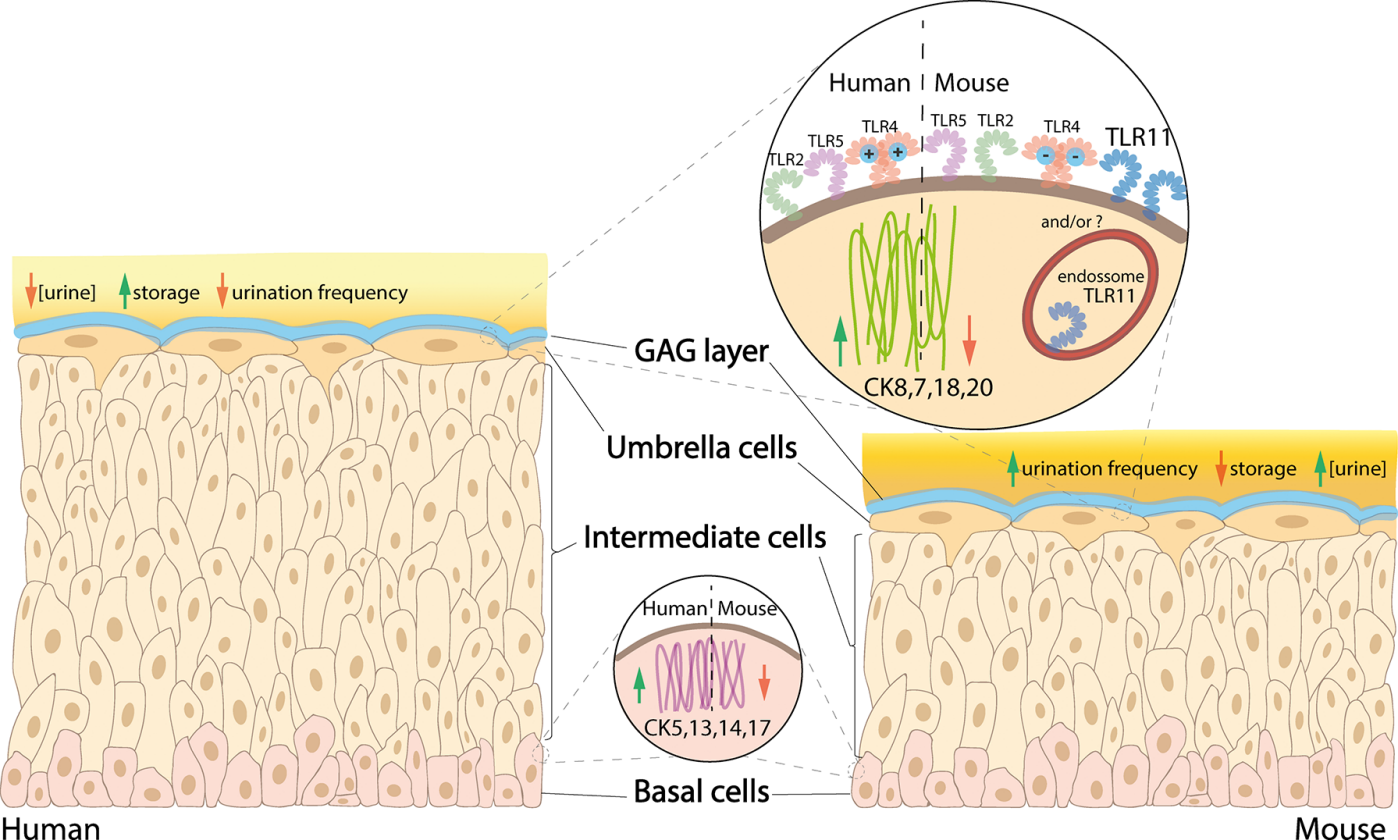
Comparison between human versus mouse urothelium. In human bladder, basal cells have higher expression of CK5, 13, 14 and 17, intermediate cells are stacked in 5-7 layers (*vs* 3-4 in mice), and umbrella cells have higher expression of CK8, 7, 18 and 20, as well as a more cationic MD-2 protein associated with the TLR4, while mice have a high expression of TLR11 (which is not present in humans). Urine is less concentrated in humans, which also have higher storage capacity and lower urination frequency compared with mice.

Murine and human bladders differ in certain anatomical features and expression of biomarkers on the epithelial surface (**Figure 1**). The mouse urothelium is typically 3-4 cells thick, making it thinner compared with human, which is 5-7 cells thick. The difference is due to the number of intermediate cells (Khandelwal et al., 2009), which could create differences in physiology and microenvironment between the upper and lower intermediate cells. In addition, the basal cells of the human urothelium have a higher expression of cytokeratin (CK) 5, 13, 14 & 17 compared with mice, whereas human umbrella cells have a higher expression of CK7, 8, 18 & 20 (Laguna et al., 2006).

As the upper intermediate cells are more competent to differentiate into umbrella cells compared with the basal-like lower intermediate cells, there may be increased expression of umbrella cell cytokeratins within the upper intermediate cells compared with those below. Although studies are lacking, as mice only have 1 or 2 intermediate cell layers, it is possible they do not possess the full array of cell-specific biomarkers present in human. Studying their differences could help establish whether murine bladders can accurately recapitulate human UTI. Lui et al. found differences in CD markers in the different layers of the human urothelium, with CD271 in basal cells and CD227 in umbrella cells; however, there was no specific CD marker for intermediate cells (Liu et al., 2012). The analogous situation in murine urothelium has not been explored.

Another species difference involves the interstitial cells (IC), which are recently described specialized cells residing in the lamina propria and detrusor muscle associated with nerves (McCloskey, 2010). Yu et al. found a high similarity in single-cell types between human and mice bladders, but single-cell transcriptomic mapping highlighted two novel IC expression types specific to human bladders, namely *ADRA2A^+^* and *HRH2^+^*. These ICs may play a role in allergic reactions and nerve conduction (Yu et al., 2019). Gavaert et al. also reported a difference between mouse and human IC, with human ICs manifesting increased contractile microfilaments, versus a fibroblast phenotype in murine ICs (Gevaert et al., 2017). The role of ICs in the host response during UTI is unknown but could inform new therapeutic targets.

Urodynamics and voiding patterns also are different between humans and mice. Both sexes of 10-week-old mice void more frequently than humans, ~10 times a day vs ~6 times a day (Chung and van Mastrigt, 2009; Aizawa et al., 2013), and also more frequently at night (Ito et al., 2017). Unlike animals over 3 kg, including humans, which possess a scalable urinary capacity and similar voiding durations, Yang et al. reported that mice scarcely store urine at all, which would affect the shape and stretching parameters of their bladders (Yang et al., 2014). Contraction of the detrusor muscle in mammalian bladders usually depends on activation of muscarinic-3 (M3) receptors, whereas mice use activation of muscarinic-2 (M2) receptors *via* an indirect mechanism (Ehlert et al., 2005; Zhou et al., 2010), which could be important for accurate disease modeling. Age may also play a role; e.g. differences were reported in voiding and storage with 12 week “mature” mice compared to “aged” 27-30 month C57BL/6 mice, with more severe bladder dysfunction in aged male mice compared with female. M3 receptor expression was downregulated in aged male mice, whereas β2-adrenoceptor was downregulated in aged females (Kamei et al., 2018). As aging is a risk factor in developing UTIs (Foxman, 2010) yet younger mice are mostly selected for UTI research, it is possible that youthful mouse models are inaccurate for modeling UTI in our aging population.

#### Host Pathogen Interactions and Immunological Differences

Humans and mice also differ in some innate immunological parameters. In UTI, the innate immune system is particularly important for host recognition of pathogen-associated molecular patterns (PAMPs) *via* pathogen recognition receptors (PRRs). PRRs recognize pathogens and help regulate a quick pro-inflammatory immune response, the mechanisms of which have been extensively reviewed (Takeuchi and Akira, 2010; Abraham and Miao, 2015). The predominant group of PRRs are Toll-like receptors (TLRs). Differences in TLR structure and function between humans and mice could influence how the innate immune system responds to a particular UTI pathogen, which could influence model accuracy.

There are 13 members of TLRs and between humans and mice; TLR1-9 are present in both, TLR10 is found in only in humans and TLR11-13, only in mice (Behzadi and Behzadi, 2016). In the urothelium, TLR2, 4, 5 and 11 (in mice) are the most competent TLRs against UTIs (Song and Abraham, 2008; Behzadi and Behzadi, 2016).

TLR4 is the most well-studied of the TLRs. TLR4 forms complexes with CD14 and MD-2 which help bind bacterial lipopolysaccharide (LPS) to initiate signaling for innate immune response (Vaure and Liu, 2014). LPS is displayed abundantly on the outside of Gram-negative bacteria such as UPEC, highlighting the importance of bacterial TLR4 interactions in the urinary tract. Despite TLR4 being present in both species, noticeable differences exist (reviewed by Vaure and Liu, 2014), with only a 57% similarity between their MD-2 proteins; this makes human MD-2 more cationic than mouse MD-2 which further affects the activation of TLR4 signaling (Vasl et al., 2009). The hypervariable region of TLR4, where MD-2 complexes with TLR4, has only 48% similarity between humans and mice, which again might lead to differences in how the host responds to infections. To date, the impact of these differences in TLR4 in human and mouse UTI has not been investigated.

TLR2, expressed in both species, has been shown to be a signaling receptor for bacterial peptidoglycan, important for recognition of Gram-positive bacteria (Takeuchi et al., 1999), and its ability to form heterodimer complexes with TLR1 and 6 broadens its spectrum to recognize other PAMPs (reviewed in Behzadi and Behzadi, 2016). TLR5 and 11 are similar in that they recognize UPEC PAMP flagellin (Hatai et al., 2016). As TLR11 is only expressed in mice, mice might have a higher capacity to recognize flagellin from UPEC, which should be kept in mind when studying this bacterial parameter.

#### Bacterial Differences

Bacterial phenotypes differ between mouse and human infections, likely influenced by differences in environment, bacterial development and growth. For example, the importance of some bacterial virulence factors seems to be completely different between human cell lines and animal models (Alamuri et al., 2010). A recent paper explored the gene expression of three UPEC strains isolated from cases of uncomplicated UTI; gene expression between human and mouse UTI was highly correlated, but 5.4% of analyzed genes were differentially regulated with 30 genes upregulated and 145 downregulated in human UTI (Frick-Cheng et al., 2020). Most of the downregulated genes were involved in anaerobic metabolism, so the authors hypothesized that the human bladder is more oxygenated than the murine. The presence and full effect of this physiological difference on bacterial phenotypes has not been explored.

Species differences in urine composition might also contribute to differences in bacterial behavior. Mouse urine has been shown to change the gene expression of UPEC when compared with human urine (Hagan et al., 2010). This study found that type 1 fimbrial genes – essential for bacterial adherence in murine models – were not expressed in 6 of 8 clinical isolates in human urine, suggesting that murine models impart a different expression phenotype. Mouse urine is more concentrated than that of larger mammals, which can affect UPEC biofilm production and cell morphology. FliC, a virulence factor encoding UPEC flagellin, was shown to be downregulated in mouse urine and upregulated in human urine (Snyder et al., 2004; Berry et al., 2009). On the other hand, filamentation, which is important for UPEC virulence, occurred in both highly concentrated human and mouse urine (Klein et al., 2015).

Murine models have been used to study antibiotic efficacy. Chockalingham et al. infected immunocompetent Balb/C mice with UPEC strain CFT073 to investigate resistance development against ampicillin, ciprofloxacin and fosfomycin, concluding that the mouse model was not suitable for studying resistance patterns, but could be useful for studying persistence (Chockalingam et al., 2019).

### Other Animal Models for UTI

Porcine models have recently been mooted as an attractive species to model pathogenesis of UTI. They have more conserved homology and structural motifs to humans compared with mice, which may make it a more accurate model particularly for immunological studies (Dawson et al., 2017). Thus far pigs have been mainly used for research into pyelonephritis and renal damage, especially vesicoureteric reflux and renal scarring in infants and establishment of upper UTI (Coulthard et al., 2002). However, until recently pigs had not been used to model cystitis. In 2019, Nielsen et al. reported a UPEC model of infection using a clinical isolate UTI89 to sustain an infection for up to 23 days in female pigs (Nielsen et al., 2019). Interestingly, as mentioned above, no intracellular UPEC were seen as has been widely reported for UTI89 in mice. The porcine model, as a large mammal, shares similarities in urine density and anatomy with humans (Nielsen et al., 2019). However, large animal models are particularly expensive. What’s more, the scope for genetic manipulation in pigs has yet to match that of mice (Barber et al., 2016).

The nematode *Caenorhabditis elegans* has been used for *in vivo* infection studies that include various UTI pathogens (Lavigne et al., 2008; Szabados et al., 2013; Engelsöy et al., 2019). Very recently, Hashimoto et al. demonstrated that UPEC mutants with defective iron acquisition-related virulence factors were a significant factor in survival of *C. elegans* (Hashimoto et al., 2021). However, as a model, *C. elegans* has only been useful for survival assays and cannot be explored as a physiological model as it lacks a urinary system. Zebrafish, a popular model for real-time visualization of infections, also lack a urinary system, but has recently been used to determine *in vivo* L-form switching of UPEC and its role in recurrent UTI (Mickiewicz et al., 2019).

Non-human primate (NHP) models benefit from their high similarity with humans. To our knowledge there are no papers describing an NHP model for UTI (Chen et al., 2019), but they have been used in the past for UTI vaccine studies (reviewed in (O’Brien et al., 2016) and viral studies (reviewed in (Estes et al., 2018). However, their expense, ethical issues and specialized facilities pose significant drawbacks.

In summary, animal models have enabled crucial advances, but relevant human-related models are still needed to fully understand UTI in patients. These models should provide complementary valuable insights into the host-pathogen interactions and allow the development of more human-relevant therapeutic approaches.

## *In Vitro* Human Cell-Based Models

The *in vitro* recapitulation of the human bladder environment and the reconstitution of the urothelium (or even the organ) is highly desirable because of the advantages such models present compared with animal models (see Section 3) and their potential for studying a wide range of conditions, from UTIs to bladder cancer, tissue regeneration/transplant and the effect of drugs and xenobiotics that contact the urinary tract. Three major strategies have been employed: i) cell monolayers, using commercially available cell lines, cells recovered from urine or derived from biopsies/explant cultures; ii) tissue/organ cultures from patients; and iii) 3D structures/organoids that mimic the urothelium after induced cell stratification and differentiation. An overview of the different bladder models used in UTI and non-UTI related research is presented in **Tables 2**, **3**, respectively.

**Table 2:** *In vitro* human bladder cell-based models used for UTI studies.

Model	Origin	Stretch	Flow	Urine	Agent	Additional info.	Ref.
***Cell lines (monolayers)***							
PD07i	Pediatric human bladder epithelial cells immortalized using the human papillomavirus type 16 E6/E7	N	Y	Y	*E. coli*	Flow chamber with shear stress (fluid replaced each 2 min)	(Andersen et al., 2012)
		N	N	N			(Klumpp et al., 2006; Berry et al., 2009)
		N	N	N		Organotypic raft cultures in semisolid medium	(Thumbikat et al., 2009)
		N	Y	Y		CellASIC Onix microfluidics system with M04S-03 plates	(Iosifidis and Duggin, 2020)
ATCC^®^ HTB-9	Human bladder epithelial cells 5637, from grade II carcinoma	N	N	N	*E. coli*		(Eto et al., 2006; Miao et al., 2015; Miao et al., 2017; Li et al., 2019; Wang et al., 2019; Patras et al., 2020; Li et al., 2021)
		N	N	Y	GBS*		(Tan et al., 2012)
		N	N	N			(Patras et al., 2020)
		N	N	N	*C. albicans*		(Coady et al., 2018)
ATCC^®^ HTB-1	Human bladder epithelial cells J82, from transitional cell carcinoma	N	N	N	*E. coli*		(Langermann et al., 1997)
ATCC^®^ HTB-4	Human bladder epithelial cells T24, from transitional cell carcinoma	N	N	N	*E. coli*		(Li et al., 2019; González et al., 2020)
		N	Y	N		Flow chamber with shear stress	(Zalewska-Piątek et al., 2020)
		N	N	N	GBS*		(Ulett et al., 2010; Tan et al., 2012)
		N	N	Y			
		N	N	N	LPS from *E. coli* and flagellin from *S. typhimurium*		(Smith et al., 2011)
EJ (MGH-U1)	Human bladder carcinoma						
Finite normal human urothelial (NHU) cells	Biopsies of the human ureter and bladder						
UROtsa	Primary culture of normal human urothelium (ureter), immortalized using the simian virus 40 (SV40) large T antigen	N	N	N	RNAse6 from *E. coli*	Cytotoxicity tests	(Becknell et al., 2015)
TEU-1 and/or TEU-2	Primary human urothelial cells (from ureter) immortalized using human papillomavirus type 16 E6E7	N	N	N	*E. coli*		(Klumpp et al., 2001; Klumpp et al., 2006; Billips et al., 2007; Billips et al., 2008)
SR22A	Primary bladder urothelial cells obtained from a biopsy of a patient with interstitial cystitis	N	N	N	*E. coli*		(Klumpp et al., 2006)
***3D structures***							
HTB-9 organoid	ATCC^®^ HTB-9 (5637) cells	N	N	N	*E. coli*	Cells under microgravity conditions	(Smith et al., 2006)
HBEP and HBLAK organoid	Human bladder epithelial progenitor cells and spontaneously immortalized (non-transformed) counterpart, derived from the trigone region of the bladder. Available from CellNTec	N	N	Y	*E. faecalis*		(Horsley et al., 2018)
Multilayered bladder rounded assembloid	Using normal human bladder tissue samples and stem cells; stroma components; fibroblasts; endothelial and smooth muscle cells	induced contraction of the muscle layer	N	N	*E. coli*	Bladder tumor assembloids were also created	(Kim et al., 2020)
***Others***							
Urinary tract epithelia cells	Upper and lower urinary tract urothelial cells from human urine (mixture)	N	N	N	*E. coli*	Assays performed in PBS suspensions	(Svanborg Edén et al., 1976)
Asymmetric Unit Membranes	Apical surface of human bladders	N	N	N			(Wu et al., 1996)
Bladder tissue sections	Surgical pathology and autopsy files	N	N	N			(Langermann et al., 1997)
Urothelial and squamous cells	Fresh urine from patients	N	N	Y			(Martínez-Figueroa et al., 2020)

*GBS, Group B Streptococcus, also known as Streptococcus agalactiae. Y, yes; N, no, refer to the use of urine, stretch or flow in these models.

**Table 3:** Relevant *in vitro* human cell-based models not used in UTI studies.

Model	Origin	Studies	Ref.
Urothelial cells	Bladder and ureters of patients undergoing urological operations	Xenobiotic studies	(Flieger et al., 2008)
Tissue specimens			
Exfoliated cells	Human urine		(Belik et al., 2008; Dörrenhaus et al., 2000)
Finite normal human urothelial (NHU) cells (potential for *in vitro* stratification and differentiation)	Biopsies/explants of histologically-normal bladder, renal pelvis and ureter, obtained at surgery from adult and/or pediatric patients	Development of models/characterization of bladder epithelium	(Rheinwald and O’Connell, 1985; Hutton et al., 1993; Southgate et al., 1994; Varley et al., 2005; Cross et al., 2005; Southgate et al., 2007; Rubenwolf et al., 2009; Rubenwolf et al., 2012; Wezel et al., 2013)
Urothelial cells	Biopsies of dysfunctional bladder		(Southgate et al., 2007)
	Urine of newborn children		(Sutherland and Bain, 1972)
	From patients with interstitial cystitis and healthy controls (stretch experiments at 20% strain)		(Sun et al., 2001; SUN and CHAI, 2004)
Urine-derived stem cells	Urine from healthy adult men		(Lang et al., 2013)
Human induced pluripotent stem cells (hiPSCs) derived from mature bladder urothelium	hiPSC line FF-PB-3AB4 established from a healthy donor’s peripheral blood mononuclear cells (PBMCs)		(Suzuki et al., 2019)
Normal human transitional cells	Ureter and embryonic bladder explants		(Reznikoff et al., 1983)
*In vitro* stratified cell layers	Biopsy of lower urinary tract from adult patients undergoing open tumor surgery		(Feil et al., 2008)
	Bladder irrigation fluids (with exfoliated cells)		(Nagele et al., 2008)
	Anatomically normal bladder and ureteral mucosa of children undergoing open kidney or bladder surgery		(Sugasi et al., 2000)
	UROtsa cell monolayers (with some cytodifferentiation), available from ThermoFisher		(Petzoldt et al., 1994; Petzoldt et al., 1995; Rossi et al., 2001)
Organ/Tissue culture	Bladder, renal pelvis and ureter of patients undergoing urological surgery with benign or no condition		(Knowles et al., 1983; Scriven et al., 1997)
	Bladder from cadavers and deceased heart-beating brain-stem dead donors		(Garthwaite et al., 2014)
3D multilayered urothelium (with cell differentiation), dynamic cultures mimicking the urine flow	Human urine-derived stem cells, urothelial cells and smooth muscle cells		(Wan et al., 2018)
Organoids and spheroids for cancer studies		Reviewed in (Wang et al., 2017; Vasyutin et al., 2019)	

As human bladder cell lines are easy to maintain in 2D and to manipulate using standard techniques, cell monolayer culture remains one of the most attractive methodologies. There are also an increasing variety of well-established human bladder cell lines that can be purchased; e.g. the American Type Culture Collection currently provides 14 certified human bladder cell lines (4 normal and 10 cancer). Those from cancer sources are among the most popular, mainly HTB-9 and HTB-4, due to their fast growth and easy manipulation. Apart from their obvious relevance for cancer studies, the data obtained with these cells in UTI research should be carefully interpreted due to the differences between normal and cancer cells. Moreover, these cells cannot acquire a native three-dimensional urothelial architecture. On the other hand, normal cells are either immortalized, which may compromise cell differentiation (Georgopoulos et al., 2011), or are derived from human samples (urine or biopsies), maintaining their ability to stratify and differentiate. In the latter case, cells can only be used during a limited number of passages so their routine use requires a large amount of material. Also, donor variation may affect reproducibility.

In general, the previously mentioned models generate one of two extremes, either highly differentiated primary cultures with lower serial growth potential, or undifferentiated cultures with higher serial growth potential. Importantly, regardless of the cell source, cells cultured in monolayers do not display growth, architecture and physiology comparable to the human urothelium, limitations that are particularly relevant to UTI (Smith et al., 2011).

In contrast, a more biologically relevant model is organ/explant culture, where intact specimens of tissues are cultured for a time. While these may maintain an accurate 3D cell architecture, stratification and differentiation status, the method is time-consuming, requires a ready availability of fresh tissue, and again, may exhibit inter-sample variability. Also, there is an increasing probability that stromal and urothelial cells will undergo unwanted mixing over culturing time, with the overgrowth of stromal cells relative to urothelial (Baker et al., 2014). Moreover, the lumen of these models is commonly exposed to air, which does not happen in the bladder *in situ*, and may affect the maintenance of cell differentiation and/or polarization (Višnjar and Kreft, 2013).

Due to the drawbacks of these strategies, other 3D experimental bladder models are emerging which attempt to biomimic the human urothelium using stem- or stem-like cells subjected to phases of propagation/expansion and stratification/differentiation. Some of them were even patented (Cross, 2003). Recently, Kim et al. reported the most complex 3D bladder multilayered model, mimicking an organized architecture of a mature organ through the assembly of a urothelium surrounding stroma and an outer muscle layer (Kim et al., 2020).

Interestingly, apart from the 3D organoid described by Horsley et al. in 2018, all the aforementioned *in vitro* models were not exposed to urine, or if so, only for a few hours, up to a day – although a few studies report the use of urine components (e.g. ammonia, urea or water). This is important, since the exposure of the apical urothelial surface to urine seems to be essential for proper differentiation and adequate mucopolysaccharide production (Horsley et al., 2018). Also, bacterial behavior and gene expression is affected by the presence of urine (Hagan et al., 2010; Reitzer and Zimmern, 2019); as urine is the natural context in which these microorganisms operate, models that do not include urine will be incomplete.

Notwithstanding the lack of a cellular immune response and the systemic host background that only *in vivo* studies can provide, it is expected that human-based *in vitro* biomimetic urothelium constructs with more physiological aspects will accelerate drug discovery for bladder diseases and even reduce the use of animal models (Baker et al., 2014). In a UTI context, promising platforms already exist for the study of host-pathogen interactions, but they lack key parameters which have a profound impact on normal bladder physiology and may dramatically influence the experimental results obtained. Below we review these key aspects and why they should be included in the next generation of models.

## Key Features of the Human Bladder That Should Be Recapitulated in Advanced Human-Cell Models, and Models That Attempt Them

Advanced *in vitro* models more closely approximating the human urothelium would be valuable for assessing the cellular changes that occur with infection. Such models would ideally incorporate at least four unique structural and biophysical aspects theorized to be important for host-pathogen interactions including: (1) tissue architecture; (2) apical urine exposure; (3) dynamic fluid flow; and (4) urothelial stretch. These key features are described in further detail below, in the context of unique bladder physiology, relevance to UTI, and current models or platforms of relevance.

### Tissue Architecture

As described above, the human bladder is lined by a unique transitional urothelium formed of approximately 5-7 layers of cells (**Figure 1**) (reviewed in Khandelwal et al., 2009). Terminally differentiated umbrella or facet cells top the intermediate cell layer and face outward into the bladder lumen in contact with urine; these unique and specialized cells are formed by fusion of intermediate layers below, are very large but flattened when stretched, and form a barrier to urine despite their highly dynamic environment. Turnover is slow under healthy conditions (on the order of weeks), but when an umbrella cell is exfoliated, the urothelium ensures replacement through the fusing and differentiation of intermediate cells, which can be replaced in turn *via* the basal progenitor layer. Umbrella cells deploy a protective apical array of asymmetric unit membrane plaques comprised of uroplakin proteins (Wu et al., 2009). The urothelium also elaborates a mucopolysaccharide-rich layer of glycosaminoglycans (GAG) which offers additional protection, enhancing the urothelial barrier against urine (Janssen et al., 2013). Indeed, the urothelium is one of the strongest epithelial barriers *in vivo* (Eaton et al., 2019) with native tissue trans-epithelial electrical resistance (TEER) values exceeding 3000 Ω*cm^2^ (Cross et al., 2005). The urothelium is not a passive barrier but is responsive to biomechanical cues, serving as a “mechanosensory conductor” as distention of the bladder stretches the urothelium during filling (Winder et al., 2014).

Given that the urothelium exhibits differential expression of specific markers throughout its layers, recapitulating the stratified human urothelium with appropriate markers and functions is critical for *in vitro* models of human bladder health and disease, and has particular relevance for UTI. The hypothesis that deep “quiescent intracellular reservoirs” in the intermediate cell layer may act as long-term reservoirs for recurrence requires a thicker multi-layer structure for studying this phenomenon. Moreover, as infection causes copious apical shedding, more than three layers are useful for retaining urothelial structure during bacterial insult. At the same time, studying adhesion properties of uropathogens, as well as innate epithelial response, ideally requires a human umbrella cell-bacteria interface.

#### Strategies to Induce or Improve Differentiated Multicellular Architecture

Much of our understanding of stratified urothelial architecture comes from animal models and human bladder biopsies (Jost et al., 1989), but recreating this architecture *in vitro* is not trivial. The following trends emerged as we surveyed the literature to elucidate what factors are critical for achieving multiple cell layers alongside umbrella cell differentiation in human-cell models.

##### Cell Source

Three-dimensional, multilayer urothelial organoid development *in vitro* has been reported using various cell sources: patient-derived cells from biopsies and surgical specimens, either by enzymatic dissociation or explant (e.g., Southgate et al., 1994; Daher et al., 2004; Cross et al., 2005; Zhang and Atala, 2013) immortalized cell lines (e.g., UROtsa used by Rossi et al., 2001); commercial primary cells or spontaneously immortalized cells (HBEP and HBLAK used by Horsley et al., 2018); human urine stem cells (Lang et al., 2013); and iPS cells (Suzuki et al., 2019). However, not all recapitulate the proper stratification and necessary hallmarks of the apical-most layer of umbrella cells, nor were used for infection. Most models report at least some important features, such as uroplakins, cytokeratins, and/or junctions, and include some form of visual evidence of a multilayer structure (e.g., electron or confocal microscopy). It remains unclear whether one cell source is superior to another, since there is no standard approach to urothelial differentiation either in terms of method or assessment. The ideal cell source would be readily available (either commercially and/or through well-described isolation techniques) and lead to reproducible differentiation in culture under defined conditions.

##### Organoid vs. Spheroid Culture

Although there is some confusion about the terms, organoids are usually defined as *in vitro* culture models that replicate features of native tissue organization, whereas spheroids are a specific, spherical form of organoid (Fang and Eglen, 2017; Vasyutin et al., 2019). While each approach has its advantages and limitations, a flattened organoid approach may be more desirable for UTI models, as the lumen of spheroid cultures would be difficult to access for inducing infection and readouts of interest beyond imaging (e.g., secreted factors, barrier function assays). Flattened organoids, on the other hand, can establish 3D tissue architecture on a 2D surface allowing for ease of infection, imaging, sampling of media, barrier function (permeability assays or TEER), and the addition of other biophysical cues such as flow and/or stretch as described below.

##### Manipulation of Soluble Factors and Media Additives

It is well-established that “high” calcium (2-4 mM) is a major factor for *in vitro* stratification and differentiation of human urothelial models (e.g., (Southgate et al., 1994; Rossi et al., 2001; Daher et al., 2004). Other additives have been used, such as FGF-7 (Tash et al., 2001), FGF-10, PPAR-γ agonists, and/or EGFR inhibitors (Suzuki et al., 2019). Urine exposure may also be required for umbrella cell differentiation (Horsley et al., 2018). Additional systematic studies of necessary factors may facilitate convergence towards a standardized media for urothelial models.

##### Culture Platform

Beyond the liquid environment, the solid substrate on which cells are cultured can greatly influence 3D tissue architecture. Substrate mechanical properties have been shown to strongly affect cell structure and function, which may influence the optimal choice of materials (Janmey et al., 2020). The most prevalent platforms have been tissue culture plastic (e.g., well plates) and Transwell^®^ inserts or similar porous membrane culture systems. The pore size typically used, when reported, is 0.4-0.45 µm diameter. Pore size and pore density may be critical factors in urothelial stratification and differentiation, but to our knowledge have not been systematically studied. Stratification is enhanced on porous membrane substrates, leading to increased urothelial tissue thickness and more uniform stratification (Cross et al., 2005; Suzuki et al., 2019) compared with well plates. In addition to providing nutrients to both sides of the tissue which could allow thicker layer formation, polarization can be achieved by providing differential cues on each side of the microporous membrane. Thus, a microporous substrate may be a requirement for advanced 3D urothelial organoid models. However, Transwells and similar platforms are static cultures not readily adaptable to dynamic biomechanical cues such as fluid flow and stretch. These advanced platforms will be described later.

##### Extracellular Matrix

The extracellular matrix (ECM) can influence cell adhesion, proliferation, differentiation and function (Hynes, 2009; Yue, 2014; Chaudhuri et al., 2020). Collagen IV is the ECM coating of choice across multiple models, and bestowed accelerated outgrowth of urothelial explants compared with laminin or fibronectin (Daher et al., 2004). Beyond substrate coatings, more complex ECM scaffolds may be beneficial for 3D urothelial model development. The Atala lab has performed extensive studies of decellularized scaffolds, including the bladder, and has described several approaches to 3D urothelial culture on ECM scaffolds (Zhang and Atala, 2013). Such scaffolds are naturally porous and provide more complex environmental cues such as multiple ECM components and the topography present in native tissue. Whether decellularized bladder or similar scaffolds enhance urothelial stratification and differentiation compared with other culture substrates remains to be determined.

##### Co-Culture

While many urothelial models are derived from a single cell source, some studies have co-cultured other cell types. The most common addition is fibroblasts (Vasyutin et al., 2019), although endothelial cells (Sharma et al., 2021) and smooth muscle cells have also been used (Zhang and Atala, 2013). Additional cell types found in the underlying stroma may provide important cues that influence urothelial proliferation, architecture and differentiation (Southgate et al., 1994). A challenge with co-culture is the inevitable increase in biological variability; alternative approaches such as defined soluble factors may be preferred when cross-talk between cell types is not critical to the study.

### Apical Urine Exposure

The urine microenvironment is an important consideration for UTI models from both the host and pathogen side. To our knowledge only two studies report the use of long-term urine exposure (>24 h) in urothelial models. One model implemented commercially available cells (primary HBEC and spontaneously immortalized HBLAK), differentiation media, and pooled urine in Millicell Transwell inserts (Horsley et al., 2018). Urine was introduced into the insert (apical side of the cell culture) 24 hr after initiating differentiation of the confluent monolayer and maintained for several weeks. The authors reported that urine was necessary for stratification, differentiation of the umbrella cell layer and GAG elaboration. This model was also used to study infection with *Enterococcus faecalis*, resulting in urothelial sloughing and formation of intracellular colonies previously observed in human patient cells. Second, a recent pre-print described a bladder-on-chip system with human bladder epithelial cells, bladder microvascular cells and neutrophils in the Emulate microfluidic platform with flow and mechanical stretch (Sharma et al., 2021). The bladder epithelium was exposed to diluted urine in the co-culture, including during infection. However, the effect of urine on model development and infection was not explicitly studied. In another recent example, a microfluidic platform with the human bladder epithelial cell line PD07i was used in experiments to infect UPEC with short-term (20 h) urine exposure under flow (Iosifidis and Duggin, 2020).

Given that few human urothelial models use urine, much remains to be learned about this key variable. *In vivo*, the umbrella cells are exposed to urine and form a tight barrier against it, while being nourished by the underlying vasculature and other cells/tissue in close proximity. Although urine may be necessary for umbrella cell differentiation *in vitro*, it is a harsh environment and cannot be the sole fluid used, even short term. For an ideal *in vitro* model, the apical surface of the urothelium would be exposed to urine while the basolateral compartment would be supported by an appropriate differentiation media. This is why platforms incorporating a microporous substrate with apical and basal compartmentalization will be more suitable for advanced urothelial models. The simplest implementation would be a Transwell^®^ or similar permeable membrane systems, such as described by Horsley et al., 2018. Other parameters to consider are the timing of urine introduction, acclimation to increasing concentrations of urine over time, and donor characteristics (sex, age, any diseases or conditions that may alter urine composition). More advanced models may implement platforms with fluid flow, such as systems described in the following sections.

Although existing static organoid models can capture some of human urothelial physiology, many biological questions probably can be answered only with a model incorporating additional biophysical aspects, including fluid flow and stretch, which are present in the human bladder and likely affect both normal physiology and infection dynamics. *In vitro* models that incorporate flow and/or stretch have been developed, but very few for the human bladder. We describe below how flow and stretch are important for both normal bladder function and UTI, and how incorporating these biomechanical cues into an *in vitro* model would be a valuable addition.

### Dynamic Fluid Flow

#### Fluid Flow in the Bladder

Given the shape and compliance of the bladder, fluid flow during voiding is extremely complex and non-uniform. Computational fluid dynamic (CFD) simulations of flow in the bladder-urethra system revealed that the peak shear stress experienced by cells is approximately 3 dyn/cm^2^ (Jin et al., 2010) – a moderate level compared with that found in the vascular system, for example. However, this level is only encountered by cells in the urethra, whereas the bladder umbrella cells experience extremely low shear, perhaps well below the levels required to activate shear-induced bacterial adhesion mechanisms. While this does not discount the hypothesis that flow-induced bacterial adhesion plays a role in bladder UTI, it does suggest that umbrella cells are perhaps less affected by this process *in vivo*, although it may be a likely mechanism in the urethra. A CFD-based study by (Ateşçi et al., 2014) produced similar results but in terms of velocity profiles within the bladder and urethra. Although this simulation data cannot be directly extrapolated to assign shear stress values experienced at the bladder wall by umbrella cells, it indicates that it is lower than in the urethra.

Aside from fluid flow possibly affecting uropathogenic bacterial adherence, it may also influence other virulence behaviors including the propensity of UPEC to take on a filamentous form that increases virulence, adhesion, invasion and escape from immune surveillance (Justice et al., 2006; Andersen et al., 2012). This may be particularly relevant for biofilm formation (Weaver et al., 2012). In addition, a number of studies have shown that microfluidic flow provides physiological cues that guide tissue architecture; as one example, in an organotypic kidney model, fluid flow altered cell shape, protein expression and transport to better approximate *in vivo* organization (Jang et al., 2013). Although currently under-studied in the context of bladder cell culture models, flow even with low shear stress could be an important cue to enhance urothelial organoid development *in vitro* as well as subsequent studies of infection. Models of the urethra with higher shear could provide insights into mechanisms of bacterial adhesion and how uropathogens migrate into the bladder. Examples of models incorporating fluid flow are provided below.

#### Models Incorporating Fluid Flow

Fluid flow has been incorporated into numerous *in vitro* model organ systems as a means for fluid and nutrient exchange, waste removal, to maintain cellular survival and tissue architecture, or to induce cellular differentiation (Baudoin et al., 2007; Carraro et al., 2008; Fritsche et al., 2009; Douville et al., 2011; Cheng et al., 2012; Park et al., 2012; Agarwal et al., 2013; Kim and Ingber, 2013; Torisawa et al., 2014; Zhang et al., 2014; Henry et al., 2017; Kasendra et al., 2018; Shin et al., 2019; Sidar et al., 2019; Azizipour et al., 2020). Many liver and kidney models use fluid flow to maintain tissue structure, deliver drugs, measure reabsorption, or carry out metabolites for ADME-tox studies (Sin et al., 2004; Sivaraman et al., 2005; Kane et al., 2006; Lee et al., 2007; Chao et al., 2009; Mahler et al., 2009; Toh et al., 2009; Jang and Suh, 2010; Novik et al., 2010; Snouber et al., 2012; Legendre et al., 2013; Esch et al., 2015; Lee et al., 2017; Maschmeyer et al., 2015; Vedula et al., 2017; Zhang et al., 2017; Bale et al., 2019; Homan et al., 2019; Jalili-Firoozinezhad et al., 2019; Tan et al., 2020). Disrupted flow patterns have been applied to induce damage that mimics tissue injury in lung and heart models (Huh et al., 2007; Giridharan et al., 2010; Huh et al., 2010; Khanal et al., 2011; Tavana et al., 2011; Nguyen et al., 2015), and microfluidic flow systems have been created to study muscle contractility (Grosberg et al., 2012), endothelial vascularization (Shin et al., 2004), pulmonary thrombosis (Jain et al., 2018), tumor-vascular invasion (Nguyen et al., 2019), blood-brain barrier (Booth and Kim, 2012), nerve injury (Park et al., 2013; Tsantoulas et al., 2013) and neuronal networks (Shi et al., 2013; Xiao et al., 2013; van de Wijdeven et al., 2018).

Microfluidic lung and gut models have also been used to study infection by bacteria and viruses. For example, fluid flow, either alone or together with cyclical strain, enabled not only proper tissue differentiation but also enhanced invasion by the infecting organism (Zhu et al., 2009; Kang et al., 2015; Barr et al., 2015; Benam et al., 2016; Villenave et al., 2017; Ortega-Prieto et al., 2018; Sunuwar et al., 2020; Tang et al., 2020; Thacker et al., 2020; Baddal and Marrazzo, 2021). Fluid flow led to upregulated invasion of colonic epithelium by Shigella bacteria (in the presence of peristalsis-like mechanical strain) (Grassart et al., 2019), increased epithelial barrier function in a lung epithelium/macrophage influenza virus co-infection model (Deinhardt-Emmer et al., 2020), and enhanced infection of alveolar or airway epithelial cells by SARS-CoV-2 (Si et al., 2020; Zhang et al., 2021). Finally, Kim et al. used a microfluidic gut chip to study the contributions of the microbiome to intestinal pathophysiology and showed that flow plus peristalsis-like mechanical deformations were necessary to protect against bacterial overgrowth reminiscent of inflammatory bowel disease (Kim et al., 2016); other studies have used microfluidic systems to investigate host-microbe interactions (Kim et al., 2012; Shah et al., 2016) and microbial diversity (Tovaglieri et al., 2019; Jalili-Firoozinezhad et al., 2019).

Despite its potential relevance, few *in vitro* bladder models have incorporated flow. One study used a Cellix chip to show that flow of UPEC in cell culture media enabled their adhesion to vascular endothelial cells (mimicking blood-borne dissemination) but had minimal effect on binding to bladder epithelial cells compared with static conditions (Feenstra et al., 2017). However, this study was performed in 2D culture in the absence of urine, and the primary goal was to investigate blood-borne dissemination of UPEC rather than invasion into bladder cells per se. Another study demonstrated filamentous rod formation and secondary infection of bladder cells by UPEC under conditions of urine flow using a flow-cell chamber (Andersen et al., 2012). The degree of filamentation was dependent on urine concentration, supporting the idea that flow can affect the infection behavior of UPEC in human cells. However, infection was tested in bladder cell cultures, not a fully differentiated urothelium. As mentioned above, a microfluidic platform with urine flow was used to study UPEC behavior in a human bladder epithelial cell line (Iosifidis and Duggin, 2020). In a recent pre-print, Sharma et al., 2021 reported a bladder-on-chip incorporating fluid flow, mechanical strain and urine exposure applied to differentiated bladder cell monolayers co-cultured with microvascular cells (see Section 5.4.3) (Sharma et al., 2021). Finally, attachment of *E. coli* to bladder cell line monolayers was found to be highest at low shear stress, allowing for maximal initial attachment of bacterial Fim H receptors *via* a slip-bond mechanism (Zalewska-Piątek et al., 2020). Thus, how the low level of shear experienced by umbrella cells *in vivo* (Jin et al., 2010; Ateşçi et al., 2014) affects their physiology and interaction with invading pathogens remains unclear. The above studies suggest the potential for uncovering further benefits of flow in UTI bladder models, including (but not limited to) maintenance of bladder epithelial differentiation and promotion of bacterial-epithelial interactions in a more physiological context.

#### Platforms/Strategies to Study the Effects of Flow

To study the role of fluid flow in UTI, the ideal platform would provide user control over flow (for example, to mimic urination patterns, to provide perfusion or nutrient replenishment); have the potential to include stretch or other biophysical and biomechanical cues (e.g., ECM); maintain two fluid compartments to aid polarization of the urothelial organoid; incorporate capabilities to interrogate the tissue using various assays; and ideally scale to a throughput allowing experimental replicates while also accommodating multiple variables such bacterial strains and therapeutic dosing. Microfluidic-based approaches such as “microphysiological systems” or “organ-on-chip” platforms have been deployed extensively for other tissue models (recent reviews include (Zhang et al., 2018; Low et al., 2020; Peterson et al., 2020) and could be an equally promising strategy for UTI models. Bacterial biofilms and antibiotic therapies have been studied in cell-free systems with flow, where flow within a microfluidic configuration allowed analysis of biofilm growth and infection potential (Terry and Neethirajan, 2014; Wright et al., 2015). As described above, bacterial infection of human cells has also responded to flow in an *in vitro* system capable of stretch (Grassart et al., 2019). Such systems demonstrate the utility and impact of flow on bacterial infection *in vitro*, but have yet to be merged with throughput. Given the timeframe of urothelial organoid differentiation *in vitro* (2-3 weeks), throughput will be essential for advanced models seeking to study multiple variables or for drug discovery efforts. Flow has been employed in high throughput, although not in an infection context, and the flow mechanism in those studies afforded limited control of flow parameters (Vormann et al., 2021). Pump-controlled flow has been deployed in high throughput in a microfluidic system with two fluid compartments for a liver model (Tan et al., 2020), but the system lacked a stretch component. A logical next step would be to incorporate flow into a high throughput system, while retaining the ability to actuate stretch for a two-fluid compartment system for advanced UTI models.

### Urothelial Stretch

Many tissues in the human body undergo modest dynamic mechanical deformation, but the bladder expands drastically during the micturition cycle. Unlike the heart and blood vessels which are constantly under pressure from the blood, the bladder experiences the extremes of being completely empty and completely full, with maximum volume capacity of the human bladder ranging from 300-500 ml. Pressures experienced within the bladder range from 0-34 cmH_2_O (0-25 mmHg) while filling and can be as high as 200 cmH_2_O (100-150 mmHg) when full (Wolfe et al., 2015). *In vivo*, pressure and stretch are coupled by the physiological processes of gradual urine filling, storage and sudden emptying, but these biomechanical cues can be studied independently *ex vivo* or *in vitro*.

Given the ability of the urothelium to increase its surface area in response to pressure or stretch, proscribing a mechanical strain experienced by the tissue is not a simple calculation, and data are difficult to obtain for humans. The urothelium can increase in apical surface area by ~50% within hours of experiencing stretch (Wang et al., 2003). Bladder tissue capacitance, a measure of umbrella cell surface area, increases over approximately 5 hr regardless of the filling rate at a pressure differential of 8 cmH_2_O (Truschel et al., 2002; Carattino et al., 2013), which represents the pressure during the extended storage phase in rabbits (Levin and Wein, 1982). Studies of rat bladders suggest than under normal filling loads, the bladder wall itself passively stretches 10-20% (Gloeckner et al., 2002). Calculations based on responses of rabbit bladder tissue to pressure suggest that umbrella cells can respond to strains in this same range (Carattino et al., 2013).

#### Biological Effects of Stretch

Cells in other mechanically active tissues have well-developed mechano-sensing properties; the bladder urothelium is no exception. Several signaling pathways and urothelial receptors contribute to bladder sensing of stretch, and are described in detail in (Janssen et al., 2017). Biomechanical stretch is known to affect a variety of cellular behaviors, such as proliferation, differentiation, maturation, and tissue-specific functions (Zimmermann, 2013; Yu et al., 2016). Limited published data exist regarding the effects of stretch on human urothelial cell behavior, although a few are highlighted below. As such, most of our understanding is derived from animal studies, typically rodent and rabbit, where species differences likely exist.

A primary urothelial function is to maintain the urine-blood barrier (Kreft et al., 2010), no small biological feat during the dramatic and dynamic mechanical fluctuations that occur with bladder filling and voiding. It has been reported that TEER initially dips and then increases during prolonged stretch (Truschel et al., 2002). Although TEER may drop with stretch, tight junctions are nevertheless maintained, as determined by immunofluorescent staining for claudins and ZO-1 and the limited permeability of tracer molecules (Carattino et al., 2013). Another group studied human urothelial cell response to various strains and noted increased proliferation under 5% strain (Gao et al., 2018).

During bladder filling and extended storage phases, umbrella cells undergo remarkable changes in surface area mediated by endo- and exocytosis (Truschel et al., 2002). Changes in these rates are triggered by mechanical stretch, but not pressure, and begin within seconds of inducing stretch at pressures as low as 2 cmH_2_O (Yu et al., 2009). This behavior was demonstrated *ex vivo* using rabbit bladder tissue by measured alterations in tissue capacitance, indicating changes in surface area, which increased up to 50% after 5 hr of prolonged stretch. Endocytosis was also remarkably upregulated with stretch as assayed by biotin-labeled membrane internalization and imaging of intracellular FITC-labeled wheat germ agglutinin. Prolonged, rather than transient or short-term, stretch is required for observed changes in surface area. However, increased endocytosis occurs in as little as 5 min after initiation of stretched conditions (Truschel et al., 2002).

Another study showed that mechanical stretch associated with filling and voiding increased the endo/exocytosis behavior of bladder epithelial cells, which use fusiform endocytic vesicle transport to increase and decrease surface membrane area during bladder expansion and contraction, respectively. This vesicle trafficking is of considerable interest because it is hypothesized to be a mechanism hijacked by certain bacterial strains that form intracellular colonies (Bishop et al., 2007).

#### Models Incorporating Urothelial Stretch

Despite the critical physiological relevance, an integrated stretch platform to support complex differentiation of urothelial organoids along with fluidics and polarized urine exposure has only begun to be explored. But at a more basic level, researchers have used multiple strategies to study stretch in urothelial cells, ranging from whole bladder distension to urothelial monolayer culture on commercially available stretch platforms. Most such studies have focused on the impact of mechanical stretch on ATP release, though few have evaluated human cells. ATP activity is linked to exocytosis and endocytosis of the mucosal surface through binding of umbrella P2 receptors; thus, the impact of stretch on ATP secretion inevitably affects membrane transport mechanisms (Wang et al., 2005) which, as pointed out previously, is potentially important for uropathogen invasion.

Tanaka et al. used an organ bath to demonstrate the impact of stretch on ATP and prostaglandin E_2_ release, which was dependent on volume-based rat bladder distension (Tanaka et al., 2011). To interrogate the impact of stretch on ATP exocytosis, Mochizuki et al. developed elastic silicone chambers mounted on glass coverslips seeded with primary mouse urothelial cells for simultaneous stretch and *in situ* Ca2+ imaging. They demonstrated that the TRPV4 cation channel mediates the release of ATP and the influx of Ca2+ *via* a stretch-dependent mechanism (Mochizuki et al., 2009). This stretch methodology was also subsequently used to analyze mechanosensation in the bladder through piezo channels (Miyamoto et al., 2014).

The importance of stretch on barrier function and tight junction proteins was demonstrated by mounting circular excised sections of rabbit urothelia in modified Ussing chambers and utilizing hydrostatic pressure-induced stretch (Truschel et al., 2002; Carattino et al., 2013). Multiple studies used a similar system to study the polarization of stretch-induced ATP secretion, demonstrating ten times more concentrated ATP supernatant on the mucosal surface versus the basal surface in rabbits (Yu, 2015). Although these models are fascinating, all results discussed thus far have been in non-human urothelial cell models and tissues.

A small subset of studies focusing on patient samples has demonstrated that mechanical stretch also affects primary human urothelial cells cultured *in vitro*. Using commercial stretch platforms, primary human cells isolated from patients with interstitial cystitis (IC), a chronic disease of unknown etiology, were shown to have significantly higher supernatant levels of ATP under stretch compared with stretched healthy control samples (Sun et al., 2001). Similarly, stretched IC patient cells demonstrated higher levels of purinergic receptor subtype P2X3, which plays a role in transmitting pain signals to the central nervous system (SUN and CHAI, 2004). This finding supports the hypothesis that urothelial cells can phenotypically mimic sensory neurons, with this phenotype driven by the presence of mechanical stretch. Therefore, primary human urothelial cells in stretch models can address crucial questions in urothelial biology.

In addition to the urothelium, the impact of mechanical stretch on other cell types such as smooth muscle cells (SMCs) isolated from the bladder has been explored. Bu *et al*. demonstrated that stretch-induced proliferation of SMCs correlated with the upregulation of metalloproteinases (MMPs) MMP-1, 2, 3, and 7 under 10% and 15% stretch conditions in a stretch-dependent manner (Bu et al., 2014). Other studies have explored the impact of stretch on SMCs, demonstrating the importance of this mechanical stimulus for aspects of the bladder beyond the urothelium (McDermott et al., 2013; Zheng et al., 2016; Pingyu et al., 2019).

#### Platforms/Strategies to Study the Effects of Stretch

Multiple commercial platforms provide the capability to study various *in vitro* tissues under mechanical stretch conditions, though certain parameters discussed in this review have yet to be incorporated into a platform capable of supporting a complete bladder infection model. Ideally, an *in vitro* system capable of uniform, biaxial strain within the range of 10-50% strain is desirable. To support a pragmatic and robust workflow, the ability to experiment with multiple replicates and image experimental samples *in situ* is vital; however, frequently used stretch platforms in their current forms are not likely to support the complexity of a fully differentiated bladder UTI model.

Existing commercial options achieve multiple-device throughput by implementing stretch on silicone-based substrates such as the uniaxial MCFX (CellScale) and STB-1400 (Strex Inc.) systems, achieving maximum strains of 12.5% and 20%, respectively. These companies also offer models that achieve biaxial stretch up to 20% in the XY plane, but most commercial biaxial models are only offered as single-well systems, such as the MCB1 (CellScale), or the STB-190-XY (Strex Inc.), which sacrifices throughput for the additional strain dimension. The most impressive system to date able to implement biaxial strain in relatively high throughput, the HT BioFlex^®^ (FlexCell), can actuate 24 wells at once, though the increased throughput sacrifices the maximum strain that the system can achieve, topping out at 8%. The company offers a lower throughput BioFlex^®^ model that achieves biaxial 20% strain in 6 devices simultaneously (FlexCell).

The ability to provide multi-faceted stimuli for cell-type differentiation is favorable; the C-Stretch system (IonOptix LLC) incorporates electrical stimulation alongside stretch to assist differentiation of naïve cultured cardiomyocytes. The ability to incorporate both fluid flow and stretch in these platforms would be widely beneficial for bladder and other *in vitro* models. Unfortunately, none of these systems incorporate them simultaneously, and all of the options discussed do not implement permeable substrates compatible with urine exposure differentiation strategies.

**Figure 2:**
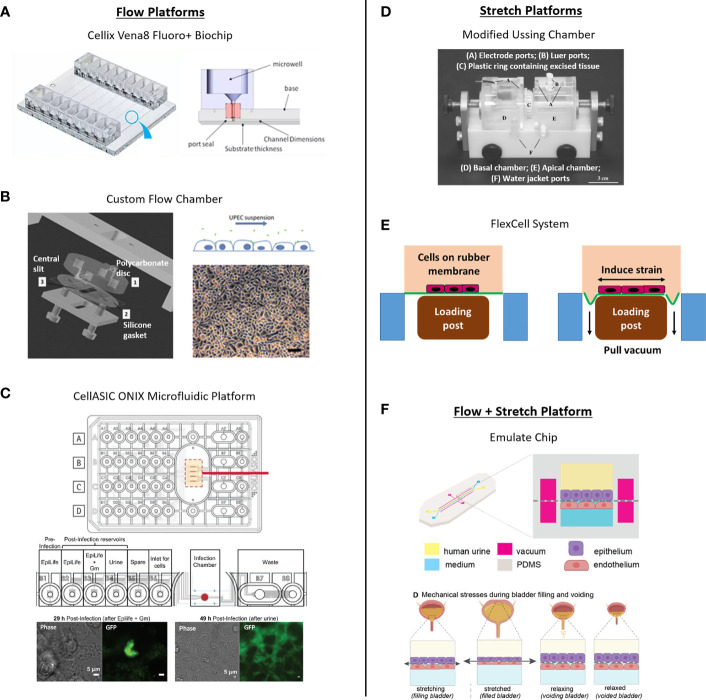
Platforms with fluid flow and/or mechanical stretch used for *in vitro* bladder studies. **(A)** The Cellix Vena8 Fluoro+ Biochip was used by Feenstra et al., 2017 to study *E. coli* adhesion to human microvascular endothelial cells and bladder epithelial cell lines. Figures modified from Cellix company website, with permission. **(B)** Custom flow chambers were used by Andersen et al., 2012 and Zalewska-Piatek et al., 2020 to study the role of flow in *E. coli* adhesion to human bladder epithelial cells. Example shown is from Andersen et al., 2012 reproduced with permission from the American Society for Microbiology. **(C)** The CellASIC ONIX platform was used by Iosifidis and Duggin, 2020 to study the role of urine composition and pH on UPEC infection of a bladder epithelial cell line. Top schematic of plate from Lee et al., reproduced with permission from Springer Nature. Bottom panel from Iosifidis and Duggin, 2020 reproduced with permission from the American Society for Microbiology. **(D)** Truschel et al., 2002 used modified Ussing chambers to induce stretch of excised rabbit bladder tissue. Figure reproduced in compliance with the Creative Commons Non-Commercial Share Alike 3.0 Uported license agreement. **(E)** FlexCell systems were used by Sun et al., 2001 and 2004 studies to investigate ATP release from primary human bladder urothelial cells from healthy and IC patients. Author schematic depicting the platform’s function. **(F)** Recently, Sharma et al., 2021 used Emulate’s organ-on-chip platform that incorporates both fluid flow and stretch in a bladder chip model of infection. Figure modified from Sharma et al., 2021 pre-print in compliance with the Creative Commons CC-BY-NC-ND 4.0 International License.

**Figure 2** depicts examples of various platforms used for *in vitro* bladder studies. To date, the closest relevant platform capable of integrating multiple mechanical stimuli is the Chip S-1^®^ system (Emulate Inc.), which utilizes microfluidic flow paths and flexible side walls exposed to vacuum to deliver simultaneous flow up to 0.3 dynes/cm^2^, and demonstrated uniaxial strain of 10%. In a recent pre-print, this platform modeled infection in differentiated HTB9 bladder cancer cell monolayers over a 6-hour voiding cycle, incorporating both flow and mechanical stretch with a porous membrane allowing for fluid transport between channels; neutrophils were recruited to sites of infection, and UPEC IBCs formed and persisted in the presence of antibiotics, supporting the hypothesis that IBCs play a role in recurrent infection (Sharma et al., 2021). Despite these strengths, areas for improvement include urothelial stratification, throughput capacity, and membrane properties, specifically pore size, pore density and drug sorption issues in PDMS (Shirure and George, 2017).

**Figure 3:**
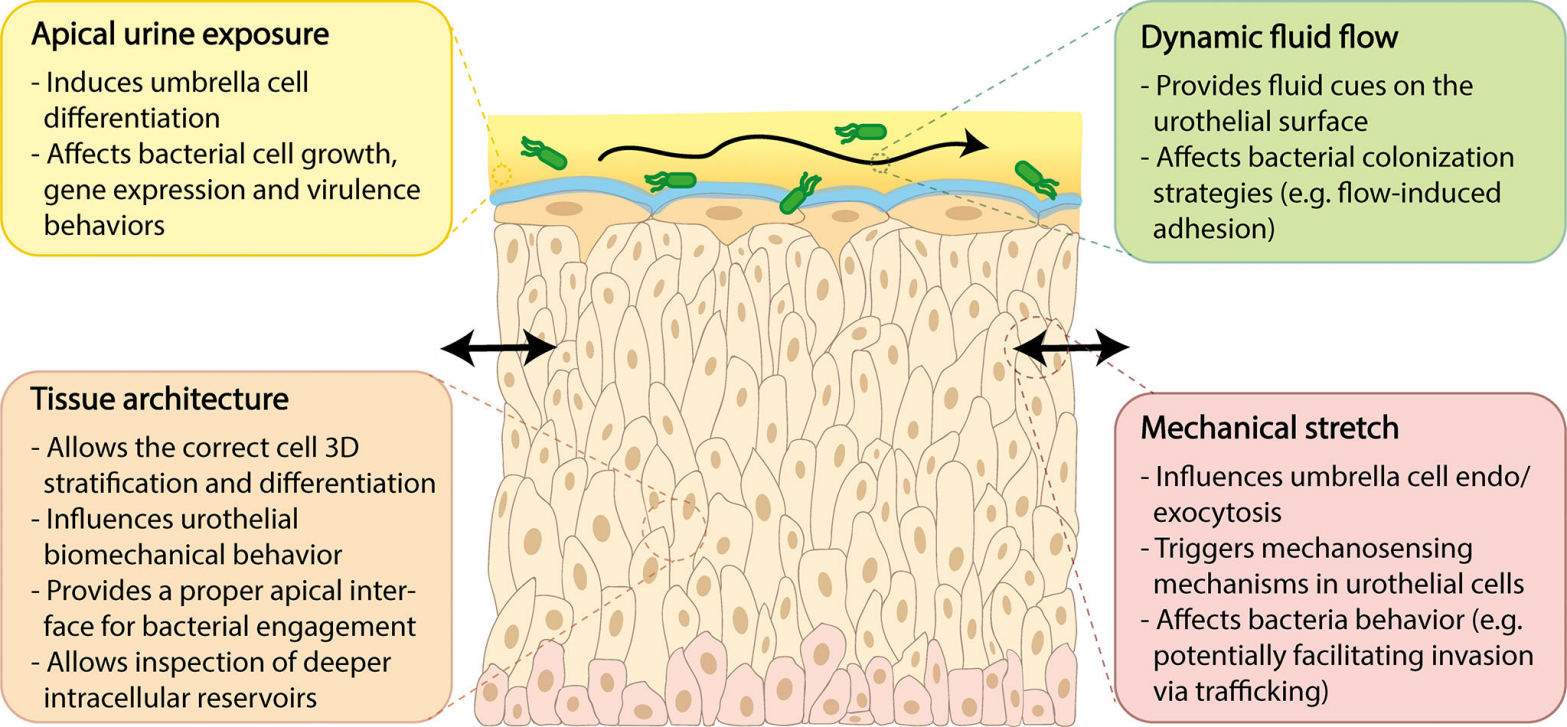
Key features required for advanced human urothelial models and their importance for UTI research.

To our knowledge the field still lacks a combined stretch- and flow-based platform with some degree of throughput to support a truly differentiated and stratified human urothelial organoid, fulfilling all the criteria necessary for exploring the complex mechanisms involved in human bladder UTIs (**Figure 3**).

## Future directions

Many mysteries still remain regarding the host/pathogen interactions of UPEC, to say nothing of the wide array of other understudied species that infect the urinary tract. On the horizon, technological advancements promise an exciting array of improvements to *in vitro* human models both generally, and for studying the infection biology of UTI in particular. The use of human cells also opens up the possibility of precision medicine by allowing the interrogation of UTI in the context of individual patients.

It is important to be aware of the limitations of *in vitro* systems. For instance, since most human cell-based models are closed structures, they still lack tissue-tissue interfaces, vascularization and circulation of immune cells. Therefore, beyond making the necessary improvements described in this review to isolated urothelial systems, we expect that linking up the bladder environment to its adjacent niches such as gut and kidney using “body-on-chip” platforms will add further nuance. Similarly, co-culturing human immune cells and/or underlying human muscle cells, fibroblasts and stroma could breathe further life into existing models. Currently, however, the recapitulation of some crucial physiology is difficult, such as the transport and/or absorption of drugs, nutrients, oxygen, inflammatory molecules and the immune cells themselves. However, significant breakthroughs have occurred recently (reviewed in Ingber, 2020). Once advanced strategies can be introduced into human-based models, such as the integration of self-organized capillary networks, a complex surrounding ECM, sensors for real-time functional measurements, and the long-term maintenance of co-cultures with different tissue types and their microbiota, the study of human pathophysiological events may well be more accurate and controllable than in animal models.

While animals will continue to reveal crucial insights about human infection biology, we must acknowledge their own limitations openly. We support a strategy whereby human cell model systems are used alongside animals to provide complementary information. It is no secret that paper referees and grant reviewers are quick to demand animal studies or to discount meticulous results gathered using even advanced human *in vitro* systems, and we quite agree with those (Ingber, 2020) who argue that the scientific community should consider whether such dampening activities are in the best spirit of scientific inquiry.

## Author Contributions

BM, CF, JR, CW and DF conceived the overall conception and design of the review. All authors contributed to writing various sections of the manuscript. All authors contributed to the article and approved the submitted version.

## Funding

This work was supported by the UK Engineering and Physical Sciences Research Council (UCL Grant Number 554581).

## Conflict of Interest

Authors CW, DF, EM, BC, and JC were or are currently employed by The Charles Stark Draper Laboratory, Inc., a not-for-profit research and development organization that develops hardware for advanced biological models. JR and BM have received research funding from AtoCap Ltd., a University College London spinoff company, to develop novel cures for urinary tract infection and bladder cancer, and JR has share options in the company. JR and CF have also received basic research funding from Pfizer.

The remaining authors declare that the research was conducted in the absence of any commercial or financial relationships that could be construed as a potential conflict of interest.
